# Characterization of a Low-Energy Cyclotron-Based Proton Beam for Preclinical Radiobiological Studies

**DOI:** 10.1016/j.ijpt.2026.101318

**Published:** 2026-04-26

**Authors:** Ana Rita C. Teixeira, Sérgio J.C. Do Carmo, Sofia Silva, Catarina I.G. Pinto, Pedro Santos, Paulo Crespo, Joana Lencart, António Paulo, Célia M. Gomes, Antero J. Abrunhosa, Filipa Mendes, Francisco Alves

**Affiliations:** 1 ICNAS/CIBIT - Institute for Nuclear Sciences Applied to Health/Coimbra Institute for Biomedical Imaging and Translational Research, University of Coimbra, Pólo das Ciências da Saúde, Coimbra, Portugal; 2 ICNAS PHARMA, Polo das Ciências da Saúde, Coimbra, Portugal; 3 Medical Physics, Radiobiology and Radiation Protection Group, Research Center of IPO Porto (CI-IPOP), IPO-Porto, Porto, Portugal; 4 Medical Physics Service, IPO-Coimbra, Coimbra, Portugal; 5 Centro de Ciências e Tecnologias Nucleares, Instituto Superior Técnico, Universidade de Lisboa, Campus Tecnológico e Nuclear, Lisboa, Portugal; 6 LIP - Laboratório de Instrumentação e Física Experimental de Partículas, Coimbra, Portugal; 7 Medical Physics Service, IPO-Porto, Porto, Portugal; 8 Departamento de Engenharia e Ciências Nucleares, Instituto Superior Técnico, Universidade de Lisboa, Lisboa, Portugal; 9 iCBR - Coimbra Institute for Clinical and Biomedical Research, Faculty of Medicine, University of Coimbra, Coimbra, Portugal

**Keywords:** Pulsed proton beam, Radiochromic film dosimetry, Radiobiology, *in vitro* research, Relative Biological Effectiveness (RBE)

## Abstract

**Purpose:**

Proton therapy is considered an attractive alternative to conventional radiotherapy in oncology, as its dose-depth curve favors tumor control while minimizing the risk of radiation-induced side effects in healthy tissue. Preclinical investigation into proton Relative Biological Effectiveness (RBE) is imperative to improve the current clinical RBE standard and subsequently optimize therapeutic efficacy.

**Materials and Methods:**

A low-energy cyclotron-based proton irradiation set-up for *in vitro* research was optimized at ICNAS-University of Coimbra. The system dosimetry was assessed using a calibration curve from a standard radiotherapy linear accelerator applied to proton-irradiated Gafchromic EBT4 films while recording integrated beam charge in real time. Pulsed dose rate measurements were performed by determination of target exposure time. As proof of concept, glioblastoma cell lines (U373 and U87) were subjected to proton irradiation for quantification of cell survival and DNA damage.

**Results:**

Homogeneous dose profiles were achieved on a 21 mm-diameter circular area at the target region for an incident proton energy of 14 MeV. A linear relation was found between proton dose at the target and integrated beam charge for pulsed dose rates from 10.8 to 16.2 Gy/s and a proton flux of ∼ 10^7^ protons/(s∙cm^2^). Proton irradiation of U373 cells yielded effects on cell survival comparable to kilovoltage X-ray exposure. U87 cells exhibited unrepaired DNA damage following proton exposure.

**Conclusion:**

A cyclotron-based pulsed proton beam was successfully optimized for *in vitro* radiobiological research, as evidenced by the first irradiation studies for evaluation of cell survival and DNA damage in glioblastoma cellular models.

## Introduction

Proton therapy (PT) is considered an alternative to conventional radiotherapy (CRT) in oncology due to its distinct dose-depth curve with a high-dose region within the tumor, the so-called spread-out Bragg peak, as well as minimal entrance and exit doses delivered to adjacent tissues. These features account for efficient tumor control while minimizing unwanted radiation damage to healthy neighboring structures, consequently reducing treatment side effects.^1^ As a result, the number of PT facilities is increasing worldwide, with more than 100 facilities in operation and over 60 planned or under construction.^2^, ^3^

In addition to the physics of PT, a more comprehensive understanding of the biological effects of proton radiation is still needed to fully benefit from its advantages. At the present time, a relative biological effectiveness (RBE) of 1.1 is used in clinical practice to estimate proton doses, suggesting a 10% higher biological outcome for PT when compared to CRT.^1^, ^4^ However, RBE might depend on multiple physical and biological variables such as dose, linear energy transfer (LET) changes within the proton dose-depth distribution, fractionation scheme, tissue type, and biological endpoints under study.^5^, ^6^ In this regard, additional preclinical radiobiological proton irradiation studies are urgently needed to improve treatment planning and increase PT effectiveness.^7^ Proton radiobiological features currently under investigation include tumor and normal tissue responses, molecular mechanisms (eg, DNA damage and repair pathways), the effects of combining proton beam delivery with chemotherapeutic, immunotherapeutic, or radiosensitizing agents and novel radiation therapy techniques (eg, ultra-high dose rates and the FLASH effect).^8^

Cyclotrons dedicated to the production of positron emission tomography radioisotopes (PET cyclotrons) can play an important role in preclinical PT research, as they are widely available for radioisotope production and often provide accessible proton beam lines for scientific research.^9^ These systems enable proton irradiation of cell cultures, small animals, and diverse materials, allowing the development, validation, and translation of PT techniques into clinical practice.^10^, ^11^, ^12^, ^13^, ^14^, ^15^ Aside from radiobiological experiments, these setups can be used for nuclear physics experiments, as well as for testing the radiation hardness of spacecraft materials and new radiation detectors.^16^, ^17^

In this work, a PET cyclotron-based external beam line has been optimized for proton irradiation of monolayer cell cultures at ICNAS, University of Coimbra.^11^, ^18^ This updated characterization includes hardware and software modifications to ensure uniform target irradiation together with dosimetric assessment with radiochromic films, quantification of dose rate (DR), and estimation of proton flux at the target. As a proof of concept, preliminary *in vitro* irradiation studies were performed in human glioblastoma cells to determine clonogenic survival and DNA damage, as glioblastoma remains a fatal condition following CRT.^19^

## Proton beam line set-up

A cyclotron-based external proton beam line for preclinical radiobiological research has been previously assembled in an 18 MeV IBA Cyclone 18/9 particle accelerator at the ICNAS facility and initially characterized by Ghithan *et al.*.^11^, ^18^ This irradiation set-up consists of a 2.4 m-long aluminum (Al) pipe, after which the proton beam is extracted to air, followed by a rotating disk with a 0.5 mm-width slit, which reduces the DR at the target to be compatible with radiobiological experiments. Further details on this “out-of-yoke” beam line are described on,^11^, ^18^ including Bragg peak measurements, proton beam current assessment using a thin aluminum (Al) foil, evaluation of optimal cyclotron magnetic field to ensure homogeneous target irradiation, and preliminary DR and absolute dose estimations.

In this work, the external line has been optimized to extract a uniform proton beam with a larger diameter to irradiate 2D cell cultures seeded in 35 mm µ-dishes (Ibidi, cat.no: 81156), whose thickness in the growth area is 180 µm, preventing significant proton energy loss. In light of this, several modifications were applied to the original irradiation set-up, including (i) the enlargement of the beam diameter to 28 mm at the end of the pipe while keeping uniformity, (ii) the introduction of a detachable collimator, which allows to the select the diameter and/or shape of the incident beam, (iii) the improvement of the current measuring device for more stable measurements, (iv) its connection to an PC electrometer (Sun Nuclear) for real-time integration of the cumulated charge measured, and (v) the incorporation of a 3D printed tailored dish holder with a 24 mm collimator at the target region to ensure uniform irradiation of the cell seeding area (21 mm diameter well).

**Figure 1:**
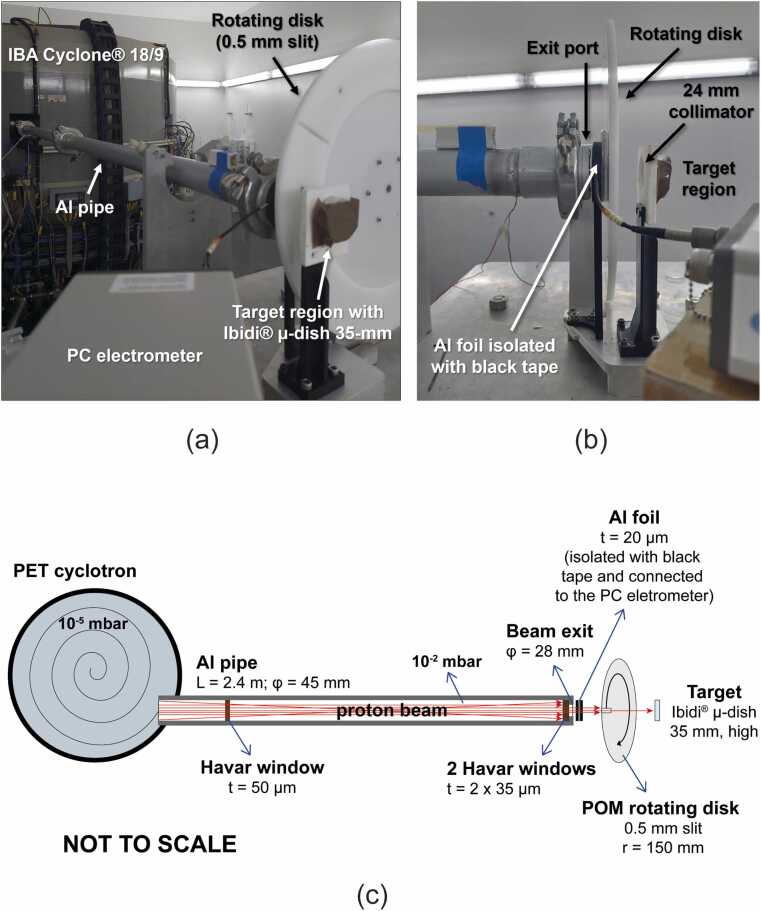
Proton irradiation set-up: (a, b) photos of the cyclotron, the Al pipe, the rotating disk, the PC electrometer, the beam exit, the Al thin foil for beam current, and the target region; (c) respective schematic representation (L: length; φ: diameter; t: thickness; r: radius).

Two 35 µm-thick Havar windows were also placed at the beam exit to enable larger beam diameters while assuring mechanical strength. The 20 µm-thick Al foil for beam current assessment in real time was carefully placed between 2 black 3903i vinyl duct tapes (3M) and the material of the rotating disk was switched to polyoxymethylene plastic to stabilize beam current measurements. Figure 1 displays the updated version of the proton irradiation set-up used in the cell irradiations reported in this work along with a diagram of all its elements.

## Estimation of beam energy at the target

After leaving the cyclotron exit port, the 18 MeV proton beam interacts with several materials before reaching the target, including Havar windows, an Al foil, polyvinyl chloride black tape, dry air, and the external surface of the polystyrene cell culture dish (Figure 1). To estimate the beam energy at the cellular layer, SRIM stopping power tables for protons (including nuclear and electronic contributions) were used to evaluate proton energy loss in each material.^20^ Table S1 included in the Supplementary Information (SI) Section 1 summarizes material thickness and density as well as total stopping power for the respective incident energies and their contribution to proton energy loss. It is noteworthy that the proton beam reaches the far end of the polystyrene dish growth area with an energy of 14.18 MeV. For the calibration process described in the next section, the beam crosses the outer layer of the film as well, which is composed of a polyester base.^21^

## Assessment of beam profile

Before dosimetric calibration and posterior cell irradiation studies, beam uniformity at the target was always verified by irradiating Gafchromic EBT4 films placed inside the cell dish, immediately after the seeding area (Figure S1). For this, multiple proton doses were delivered, maintaining the stripper current constant and increasing irradiation time. Proton-irradiated films were then scanned in an Epson Expression 10000XL scanner in landscape orientation using the 48-bit RGB transmission mode and the 72-dpi setting. Figure S2 displays an example of vertical and horizontal profiles analyzed in the Fiji software.^22^ Both profiles show a plateau corresponding to a gap of 59 pixels, that is, 20.8 mm in 72 dpi images. Thus, beam homogeneity was confirmed for a diameter of 21 mm in the EBT4 film, matching the dish growth area, allowing future quantification of target dose and subsequent cell irradiation experiments.

## Dosimetric calibration

EBT4 films are associated with higher signal-to-noise ratios and less uncertainties for the same layer composition when compared to the EBT3 model.^23^, ^24^ Thus, to obtain a photon calibration curve, 10 EBT4 films (batch 01152401, area = 5×5 cm^2^) were exposed to a 6 MV linear accelerator beam (source to surface distance SSD = 100 cm, field size = 10 x 10 cm^2^, DR = 600 Monitor Units MU/min) at a 5 cm depth within a solid water phantom (20 1 cm-thick horizontal slabs, type RW3, PTW). Additionally, an independent measurement with a Farmer ionization chamber (model 30013, PTW) at a depth of 10 cm allowed for conversion of MU in gray (Gy) as described in the IAEA TRS report no. 398.^25^ After a self-developing period of 24 h in the dark, the irradiated films along with an unexposed sample were scanned in a landscape position in the center of the scanner bed using the 48-bit RGB transmission mode and the 72-dpi setting in an Epson Expression 10000XL scanner.^26^ Square Region of Interests (ROIs) were drawn for each film image and analyzed in the Doselab Pro software (Varian Medical Systems) to obtain their optical density (OD) on the red channel, which is the most sensitive for doses up to 10 Gy.^27^ Correlating the OD values for each channel with the corresponding dose given by its MUs, a red photon calibration curve was plotted and fitted with a grade-3 polynomial function, including the axes origin.

Then, multiple EBT4 films of the same batch were placed inside the cell dish, in the seeding area, and irradiated with protons while beam current and integrated charge were recorded in real time with the electrometer. Their OD values were obtained following the same scanning and analysis method, including a square ROI inside the circular exposed region and the scanning of a non-irradiated sample to account for film and scanner non-uniformities. Each scanning session started at least 30 minutes after turning the scanner on and following a warm-up period of 5 blank scans. After this process and applying the photon calibration curves to proton ODs, we were able to establish a relationship between the integrated beam charge and the target absorbed dose.

**Figure 2:**
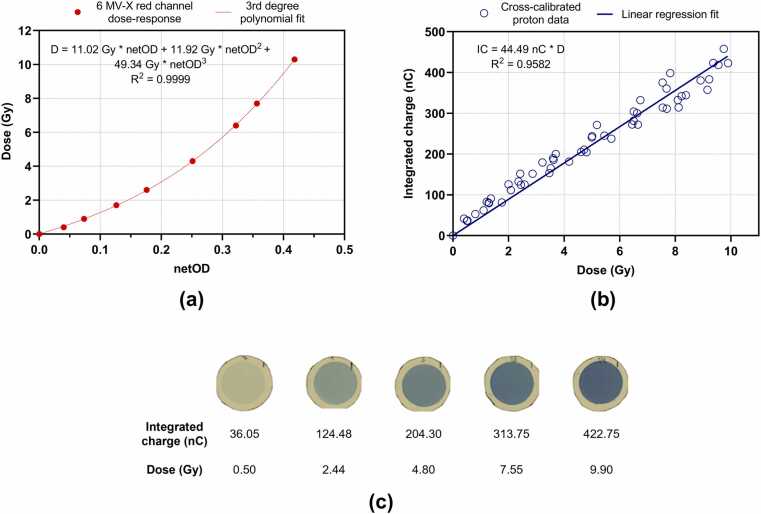
Dosimetric calibration using EBT4 films. (a) Dose-response curve for 6 MV-X photons on the red channel and respective 3rd degree polynomial fit. (b) Proton calibration curve associating the integrated beam charge in nC recorded by the electrometer with film proton dose in Gy and corresponding linear regression fit (4 independent irradiations). Polynomial and linear regression fits were calculated in GraphPad Prism 9.0. (c) Representative images of the proton-irradiated films along with respective integrated beam charges (nC) and absorbed doses (Gy).

The dose-response curve of EBT4 films for 6 MV photons on the red channel is shown on Figure 2a along with its polynomial fit. This relation between photon dose and netOD allowed us to estimate the absorbed dose from the netOD of proton-irradiated films. At the same time, beam current and integrated charge were also recorded with the electrometer for each proton irradiation. The proton calibration curve for 4 independent irradiations is displayed in Figure 2b and is valid for beam currents in the interval 1008-2371 pA and correspondent mean DRs between 0.92 and 2.88 Gy/min. The mean dose rate (MDR) is defined as the ratio of film dose (D_film_) and total irradiation time (t_total_) from beam on to beam off. All the films considered in this calibration displayed a maximum deviation to the mean dose value of 1.5% in the square ROI drawn within the plateau region. A ratio of 44.49 nC/Gy was determined for proton beam delivery. Figure 2c shows examples of proton-irradiated films along with respective live-recorded integrated beam charges and estimated absorbed doses.

## Dose rate quantification

**Figure 3:**
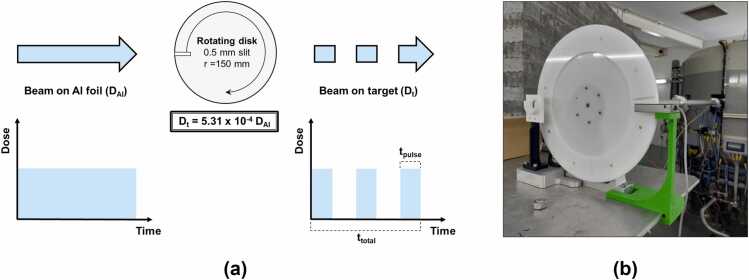
Pulsed dose rate measurements. (a) Schematic representation of the effect of the rotating disk (0.5 mm-width slit, 150 mm radius, dose reduction factor of 5.31 x 10^−4^) in the proton beam highlighting the differences between total beam delivery time ($t_{\mathrm{total}}$) and pulse duration ($t_{\mathrm{pulse}}$). (b) Photoelectric sensor positioned alongside the disk and connected to a digital oscilloscope to record pulse duration and rotation frequencies for multiple values of voltage controlled by a DC power supply.

As explained in section “Proton beam line set-up,” the proton beam is scattered by a 150 mm-radius rotating disk with a 0.5 mm-width slit, resulting in a pulsed beam reaching the target with a geometrically reduction factor of 5.31 x 10^−4^. When defining DR for this system, different irradiation times must be considered: total beam delivery time ($t_{\mathrm{total}}$), which corresponds to the time the beam is on, and pulse duration ($t_{\mathrm{pulse}}$), the time the beam passes through the slit in 1 disk rotation (Figure 3a).

To estimate the pulsed dose rate (PDR), a photoelectric sensor (ref. 903-3927, RS Amidata) was positioned alongside the rotating disk (Figure 3b) and connected to a digital oscilloscope (PicoScope 2203, Pico Technology Limited). This setup enables recording and measuring of each pulse duration ($t_{\mathrm{pulse}}$) and disk rotation frequencies (f) for multiple voltage values controlled by a DC power supply. These quantities along with the MDR enable the determination of PDR and the dose per pulse (DPP) according to the SI Section 3. In this setting, the dose per pulse (DPP) will depend on the rotation of the disk. Table 1 displays the measurements of disk rotation frequency (f) and pulse duration ($t_{\mathrm{pulse}}$) measured with the oscilloscope for several power supply voltages and corresponding PDRs and DPPs for MDR of 2.0, 2.5, and 3.0 Gy/min calculated with Eqs. S3 and S7.

**Table 1:** Disk rotation frequency (f) and pulse duration ($t_{\mathrm{pulse}}$) for multiple supplied voltages as well as respective pulsed dose rates (PDRs) and doses per pulse (DPPs) for mean dose rates (MDRs) of 2.0, 2.5, and 3.0 Gy/min.

				MDR = 2.0 Gy/min		**MDR = 2.5 Gy/min**		**MDR = 3.0 Gy/min**	
Power supply voltage (V)	f**(rpm)**	$t_{\mathrm{pulse}\;}$**(s)**	Beam opening time per minute (ms)	PDR (Gy/s)	DPP (mGy)	PDR (Gy/s)	DPP (mGy)	PDR (Gy/s)	DPP (**mGy)**
8	57.1	3.3 x 10^−3^	186.5	10.7	35.0	13.4	43.8	16.1	52.5
10	95.0	2.0 x 10^−3^	192.8	10.4	21.1	13.0	26.3	15.6	31.6
12	132.9	1.4 x 10^−3^	181.9	11.0	15.1	13.7	18.8	16.5	22.6
14	167.6	1.1 x 10^−3^	190.9	10.5	11.9	13.1	14.9	15.7	17.9
16	199.9	9.0 x 10^−4^	182.2	11.0	10.0	13.7	12.5	16.5	15.0
18	234.6	8.0 x 10^−4^	188.1	10.6	8.5	13.3	10.7	15.9	12.8
20	266.7	7.0 x 10^−4^	184.9	10.8	7.5	13.5	9.4	16.2	11.3
22	300.7	6.0 x 10^−4^	173.6	11.5	6.7	14.4	8.3	17.3	10.0
		Mean	185.1	10.8		13.5		16.2	
		SD	5.6	0.3		0.4		0.5	

For our calibration and these rotation frequencies, this irradiation system offers beam pulses with a PDR between 10.8 ± 0.3 and 16.2 ± 0.5 Gy/s (mean ± SD) and a DPP ranging from 6.7 to 52.5 mGy, while keeping the opening time per minute invariant. For the cellular irradiations described in the next sections, the disk rotation frequency was kept constant at 100 rpm. Additionally, our MDRs correspond to proton fluxes at the target ranging from 5.76 to 8.64 x 10^6^ protons/(s∙cm^2^) and at the Al foil from 1.08 to 1.63 x 10^10^ protons/(s∙cm^2^). Proton flux calculation details are presented in SI Section 4.

## *In vitro* radiobiological studies

In preliminary irradiation studies to evaluate clonogenic survival, U373 cells were seeded in 35 mm μ-dishes and allowed to adhere overnight. Different cell densities (150-2250 cells per dish) were plated considering treatment dose and previous densities used in similar photon irradiation studies.^28^, ^29^ Before irradiation, the dishes were filled to the top with culture media and sealed with parafilm to ensure that the cultures were submerged during treatment and to prevent unwanted spills while the dish was placed in a vertical position. To determine the survival curve, the cells were exposed to proton doses up to 6 Gy. Culture medium was replaced by 2 ml of fresh medium post-irradiation, and cells were incubated at 37 ºC in a 5% CO_2_ atmosphere for 10 or 11 days for optimal colony growth. Controls were subjected to the same handling except for radiation treatment. After colonies with at least 50 cells were observed, the cells were fixed with formaldehyde (1% in PBS), stained with crystal violet (0.1% in absolute ethanol), and counted manually under an inverted microscope.

**Figure 4:**
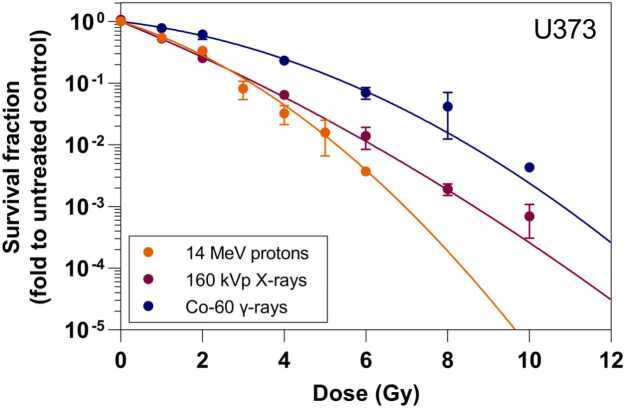
Clonogenic survival of U373 cells (survival fraction versus radiation dose) after treatment with 14 MeV protons vs treatment with photon radiation (160 kVp X-rays or Co-60 γ-rays). Survival curves for photon exposure were adapted from our previous paper by Teixeira *et al.* (2025).^29^ Solid circles represent experimental data of 2-4 independent assays (Mean ± SEM) and solid lines indicate the LQM fit to experimental values.

The proton survival curve is presented in Figure 4 fitted to the linear quadratic regression model (LQM)^30^ and benchmarked against our previously published survival curves for 160 kVp X-rays and Co-60 γ-rays.^29^ We observed that U373 cells are more sensitive to protons than to γ radiation. However, a similar clonogenicity was found for protons and kilovoltage X-rays, as no significant differences were observed between survival fractions. Estimated LQM parameters for 14 MeV protons (Table S3) confirm this trend when compared to the previously reported photon ones. In particular, a higher β component matches with a greater curvature for higher-dose protons when compared to X-rays. Analogously to photon irradiation, U373 cells irradiated with protons are also more impacted by lethal than by sublethal events (α > β). Additionally, a moderate α/β ratio of 6.63 Gy following PT was found, suggesting that dose fractionation might not be crucial for this cell line.^29^

Finally, RBE estimations (Table S3) indicate divergencies from the clinical proton standard of 1.1,^1^, ^4^ being significantly higher assuming γ radiation as reference, for both D_50%_ and D_10%_ (2.02 and 1.73, respectively). By contrast, RBE values close to 1 were found using kilovoltage X-rays as reference for survival fractions of 50% and 10% (RBE_50%_ = 0.89 ± 0.12; RBE_10%_ = 1.04 ± 0.19; mean ± SEM). Additional information on the LQM parameters as well as survival fraction and RBE calculations is available in SI Section 5.

In exploratory irradiations for DNA damage quantification using γH2AX immunocytochemistry, U87 cells were seeded in μ-dishes at a density of 325 000 cells/ml and left to adhere overnight. The dishes were filled to capacity with culture media and sealed with parafilm prior to proton exposure (5 Gy). After irradiation, medium was freshly replaced (2 mL per dish). Controls were subjected to the same handling except for radiation treatment. U87 cells were fixed at 1 and 24 hours after radiation exposure, and further details regarding the γH2AX immunofluorescence protocol are reported in SI Section 6.

**Figure 5:**
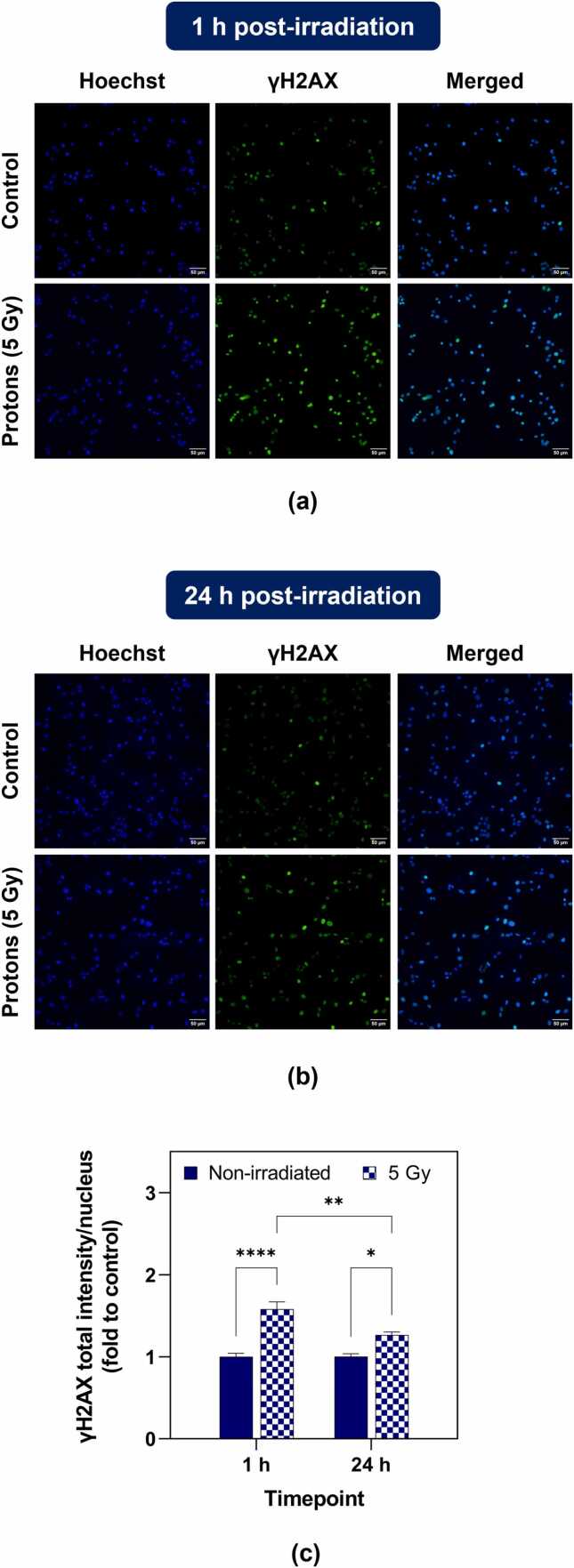
Representative images of immunocytochemistry with Hoescht (blue) and γH2AX (green) following proton treatment (14 MeV, 5 Gy) in U87 cells at (a) 1 hour and (b) 24 hours post-irradiation (20x magnification, scale bar: 50 µm). (c) Image quantification as γH2AX total fluorescence intensity per nucleus (fold to control: non-irradiated dishes at 1 hour post irradiation, Mean ± SEM). Three independent assays were performed with th imaging fields acquired per condition and a minimum of 100 cells quantified per field. Statistical differences were determined using a 2-way ANOVA followed by a Tukey’s test between treated and control conditions (* *P* < .05, ** *P* < .01, **** *P* < .0001).

Figure 5a and b display representative immunofluorescence images for 1 and 24 hours after proton irradiation (14 MeV, 5 Gy) along with control conditions. Immunostaining quantification is presented in Figure 5c as total fluorescence intensity per nucleus (fold to control). Here, controls are non-irradiated dishes fixed at 1 hour after radiation treatment. At 1 hour post-irradiation, U87 cells revealed a 58% increase in γH2AX fluorescent signal compared with untreated controls, which reduced to a 26% increase at 24 hours after PT, suggesting the presence of unrepaired DNA damage. No significant differences were observed between the non-irradiated conditions at 1 and 24 hours.

Cell culture methodology used for maintenance of U87 and U373 human glioblastoma cell lines in between radiobiological experiments is specified in SI Section 7.

## Discussion

In this work, we describe our low-energy cyclotron-based proton beam line for *in vitro* radiobiological research along with the first irradiation experiments performed using glioblastoma cellular models. In contrast to the previous configuration,^11^, ^18^ this new setting is specifically tailored to deliver a uniform proton beam to cell monolayers seeded in μ-dishes while minimizing proton energy loss. Among the several improvements made, the addition of a PC electrometer and efficient electrical insulation of important components (eg, the Al foil and the rotating disk) proved to be key aspects to ensure beam monitoring in real time and to increase current signal-to-noise.

Before the dosimetric calibration of the system, we estimated the proton beam energy immediately after the surface of the μ-dish to be 14 MeV, whose range in water is around 2 mm.^31^ This beam is suitable to irradiate monolayer cultures of glioblastoma cells localized in the pre-Bragg peak region, as their culture thickness is 18 µm according to measurements obtained from confocal microscopy.^32^ Complementarily, the beam was meticulously aligned to ensure uniform irradiation of the target area, and its profile was characterized using EBT4 films. Beam profile analysis showed 21 mm vertical and horizontal plateaus, matching the dish growth area. From a dosimetric standpoint, our beam alignment minimized uncertainties during the evaluation of film ODs and ultimately contributed to uniform dose delivery across the entire cell monolayer.

For calibration purposes, EBT4 films were placed immediately after the μ-dish surface and therefore exposed to a proton beam of 14.18 MeV in their active layer (Table S1). The mass stopping power of protons in water at the dosimetric site (13.57 MeV) is 35.70 MeV∙cm^2^/g whereas at the cells’ position (14.18 MeV) is 34.45 MeV∙cm^2^/g, resulting in a LET variation of 3.6%.^31^ Similarly, Reinhardt *et al.* (2015) also described a negligible quenching effect in EBT3 film response for LET values up to 42 MeV∙cm^2^/g.^33^ The absence of an under-dose response for EBT3 films has also been described for clinical proton beams at the entrance due to minor LET changes.^34^, ^35^ Hence, while not exact, our film positioning approach was practical and sufficiently accurate for the estimation of absorbed dose at the radiobiological target.

Further dosimetric characterization based on the application of a 6 MV calibration curve to proton-irradiated EBT4 films while recording integrated beam charge allowed us to establish a linear relationship between this quantity and the target absorbed dose, contributing to efficient dose delivery in real time during cell irradiations. The success of this cross-calibration method was guaranteed by minimizing uncertainties in film OD measurements using a well-established protocol for film dosimetry using megavoltage photon beams (including absolute calibration of the LINAC based on the IAEA TRS 398^25^) as well as by the adoption of good practices of film handling, scanning, and processing as described in the literature^26^, ^36^, ^37^ while keeping protocol consistency between photon and proton irradiations within the same film batch.

Moreover, we observed that EBT4 films maintain their linear response under these irradiation conditions. Thus, adding extra slits to the degrading disk or modifying their size would eventually lead to DR increase and enable the exploration of the FLASH effect with comparative irradiation of tumor and normal tissue cells, as well as the establishment of an upper DR limit that prevents radiochromic film quenching for pulsed proton beams.

Taking a closer look at pulse duration for each rotating frequency of the degrading disk with a slit, we showed that our irradiation system allows pulsed DRs from 10.8 to 16.2 Gy/s for mean DRs in the range 2-3 Gy/min and independent from the disk rotation frequency. Such pulsed DRs are in between those observed for CRT (< 0.1 Gy/s) and FLASH irradiation (> 40 Gy/s) and comparable to flattening filter-free stereotactic beam radiation therapy.^38^, ^39^ This result provides an opportunity to collect *in vitro* radiobiological data in this window, which is currently scarce for hadron beams.^40^

Furthermore, for this cyclotron beam line, we estimated a proton flux in the 10^6^ – 10^7^ protons/(s∙cm^2^) range, which is lined up with established European standards for radiation hardness experiments of space-qualified electronics (eg, single event effects testing).^41^, ^42^ As a result, our upgraded proton beam line is now optimized for a dual application: radiobiological *in vitro* research and the study of space radiation effects on electronic components.

As proof of concept, this work reports the first cell irradiation studies performed with our optimized cyclotron proton beam line in glioblastoma cellular models. We successfully obtained an experimental survival curve for U373 cultures subjected to 14 MeV PT. This cell line displayed higher radiosensitivity following proton irradiation when compared to the Co-60 clinical standard, which is reflected in the high RBE values estimated. However, identical clonogenicity was detected after proton and kilovoltage X-ray irradiation. Although the use of 160 kVp X-rays is unusual for RBE calculations in PT, as they exhibit significantly higher LET than megavoltage photons, these radiobiological similarities might indicate that both radiation qualities have a similar LET. Our results are consistent with LET values reported in the literature, where kilovoltage X-rays have a LET at least 10 times higher than Co-60 γ radiation and are likely comparable to 14 MeV protons in the entrance region.^15^, ^43^, ^44^ Nonetheless, LET considerations for our proton beam line still need further validation from Monte Carlo simulations. Finally, RBE dependence on biological endpoint and on the type of reference photon radiation was verified for the pre-Bragg peak region of a low-energy proton beam.

A second radiobiological experiment was performed to evaluate DNA damage in U87 glioblastoma cells. After exposure to a moderate proton dose (5 Gy), a boost in γH2AX signal intensity was detected at 1 hour post-irradiation, indicating an increase in the number of DNA double-strand breaks. Although the fluorescence declines at 24 hours after treatment, it remains significant when compared to non-irradiated conditions, suggesting the presence of unrepaired DNA double-strand break that might lead to cell impairment and eventually death. These preliminary irradiation studies highlight the feasibility of our cyclotron set-up to deliver a proton beam suitable for *in vitro* radiobiological research, namely clonogenic survival and DNA damage studies, and suitable for addressing RBE-related questions.

## Conclusion

A low-energy cyclotron-based pulsed PT set-up was successfully established for *in vitro* radiobiological studies. In particular, we have confirmed the linearity of response for a 14 MeV proton beam in the entrance region using EBT4 radiochromic films. Nevertheless, LET-related questions still need further clarification with Monte Carlo simulations.

Proton flux measurements confirmed an extra application of our beamline for radiation hardness experiments of space electronics. Furthermore, pulsed DR evaluation positioned our beam line between conventional and FLASH radiation therapy systems, offering a possibility to assess radiobiological *in vitro* data in this gap. Minor modifications in the current system would also allow the investigation of the FLASH effect in addition to setting up an upper DR limit for the absence of radiochromic film quenching.

Exploratory proton irradiation studies with glioblastoma cellular cultures for quantification of clonogenic survival and DNA damage suggest identical radiobiological outcomes to kilovoltage X-ray exposure and a higher effect than Co-60 γ radiation, although additional comparative studies with other cancer cell lines and different biological assays are still needed to confirm this trend. Lastly, the RBE dependence on biological endpoint and photon radiation quality was verified, highlighting the potential of our irradiation set-up to address RBE challenges.

## CRediT authorship contribution statement

**Ana Rita C. Teixeira:** Data curation, Formal analysis, Investigation, Methodology, Software, Visualization, Writing – original draft. **Sérgio J. C. Do Carmo:** Conceptualization, Investigation, Methodology, Validation, Writing - review and editing; **Sofia Silva:** Formal Analysis, Investigation, Methodology, Software, Validation; **Catarina I. G. Pinto:** Formal Analysis, Investigation, Methodology, Validation, Writing - review and editing; **Pedro Santos:** Methodology. **Paulo Crespo:** Conceptualization. **Joana Lencart:** Formal Analysis, Methodology, Software, Validation; **António Paulo:** Project administration, Conceptualization, Funding acquisition, Resources, Validation, Writing - review and editing; **Célia M. Gomes:** Conceptualization, Resources, Validation and Writing - review and editing; **Antero J. Abrunhosa:** Project administration, Conceptualization, Funding acquisition, Resources, Writing - review and editing; **Filipa Mendes:** Conceptualization, Funding Acquisition, Resources, Supervision, Validation, Writing - review and editing; **Francisco Alves:** Conceptualization, Funding acquisition, Resources, Supervision, Validation, Writing - review and editing.

## Data availability

Research data is stored in an institutional repository and will be shared upon reasonable request to the corresponding author.

## Statistical analysis

A. R. C. Teixeira.

## Declaration of Conflicts of Interest

The authors declare that they have no known competing financial interests or personal relationships that could have appeared to influence the work reported in this paper.

## Footnotes

Appendix A Supplementary data associated with this article can be found in the online version at doi:10.1016/j.ijpt.2026.101318.
