# Supplementary material for: Characterization of a Low-Energy Cyclotron-Based Proton Beam for Preclinical Radiobiological Studies
Source: Int J Part Ther. 2026 Apr 26;20:101318. doi: 10.1016/j.ijpt.2026.101318 (PMC13156708; doi:10.1016/j.ijpt.2026.101318)
Supplement: Supplementary file 1 — Supplementary material [file mmc1.docx]

Supplementary Information

Table of Contents

[1. Estimation of beam energy at the target 2](#_Toc219108119)

[2. Assessment of beam profile 3](#_Toc219108120)

[3. Dose rate quantification 4](#_Toc219108121)

[4. Estimation of proton flux 6](#_Toc219108122)

[5. Clonogenic survival and the LQM model 8](#_Toc219108123)

[6. DNA damage quantification 10](#_Toc219108124)

[7. Cell culture details 11](#_Toc219108125)

[References 12](#_Toc219108126)

## Estimation of beam energy at the target

SRIM stopping power tables were used to estimate proton energy loss in each material traversed by the beam and assess proton energy at the cells’ position[1]. To obtain these tables, we selected “Hydrogen” as the beam ion and included target information about each material (density and element composition). For estimating stopping power S_int_ for intermediate values of proton energy (E_int_), a linear interpolation was performed according to **Eq. S1**, where E_1_ and E_2_ are the lower and upper values of energy (E_2_ - E_1_ = 1 MeV in this case) and S_1_ and S_2_ are the respective stopping power values obtained from the tables.

|  | $S_{int}=S_{1}+\left( S_{2}-S_{1} \right)\cdot\left( \frac{E_{int}-E_{1}}{E_{2}-E_{1}} \right)=S_{1}+\left( S_{2}-S_{1} \right)\cdot(E_{int}-E_{1})$ | **(S1)** |
| --- | --- | --- |

Ultimately, the energy transmitted through the material (E_trans_) is given by

|  | $E_{trans}=E_{inc}- S_{int}\cdot t$ | **(S2)** |
| --- | --- | --- |

where E_inc_ is the incident energy and t is the thickness of the material.

**Table S1** outlines the different materials traversed by the proton beam from the cyclotron exit port to the target region along with their thickness, density and respective contribution for proton energy loss. In the calibration process, where the film is positioned after the dish growth area, the beam traverses an extra layer of polyester film base until reaching the active layer. Density and composition of Havar^®^ can be found in [2]. For dry air calculations, it was assumed a bunker temperature of 22 ºC and pressure of 81 325 Pa. For the black isolation made of Polyvinyl Chloride (PVC), the following atomic percentages were considered: 57% Cl, 38% C and %5 H. For the polystyrene (PS), atomic percentages of 50% H and 50% C were considered. Finally, density, thickness and atomic percentages of the polyester film base in EBT3 films are available in [3] (equivalent for EBT4 films).

**Table S1**

Different materials traversed by the proton beam from the cyclotron exit port to the cell dish along with their thickness, density and respective contribution for energy loss.

| **Material** | **Thickness (μm)** | **Density (g/cm^3^)** | **Incident energy (MeV)** | **Stopping Power (keV/µm)** | **Transmitted**  **Energy (MeV)** |
| --- | --- | --- | --- | --- | --- |
| Havar^®^ | 50 | 8.30 | 18.0 | 14.71 | 17.26 |
| Havar^®^ | 2 x 35 | 8.30 | 17.26 | 15.18 | 16.20 |
| Dry Air* | 9.05 x 10^3^ | 9.60 x 10^-4^ | 16.20 | 2.62 x 10^-3^ | 16.18 |
| PVC | 150 | 1.40 | 16.18 | 3.37 | 15.67 |
| Al_2_O_3_ | 20 | 3.99 | 15.67 | 10.25 | 15.47 |
| PVC | 150 | 1.40 | 15.47 | 3.49 | 14.95 |
| Dry Air* | 4.80 x 10^4^ | 9.60 x 10^-4^ | 14.95 | 2.80 x 10^-3^ | 14.81 |
| PS | 180 | 1.06 | 14.81 | 3.53 | **14.18** |
| Polyester film base | 125 | 1.35 | 14.18 | 4.39 | 13.63 |

PVC: Polyvinyl Chloride (black isolation tape); Al_2_O_3_: Aluminium Oxide; PS: Polystyrene.

* For bunker temperature T = 22 ºC and pressure P = 81 325 Pa.

## Assessment of beam profile


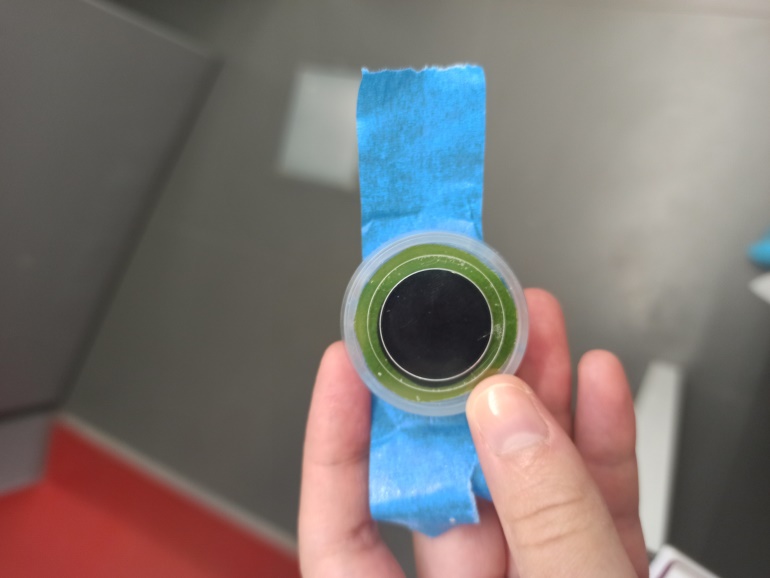


**Fig. S1.** Proton-irradiated EBT4 film inside the dish immediately after the cell growth area.


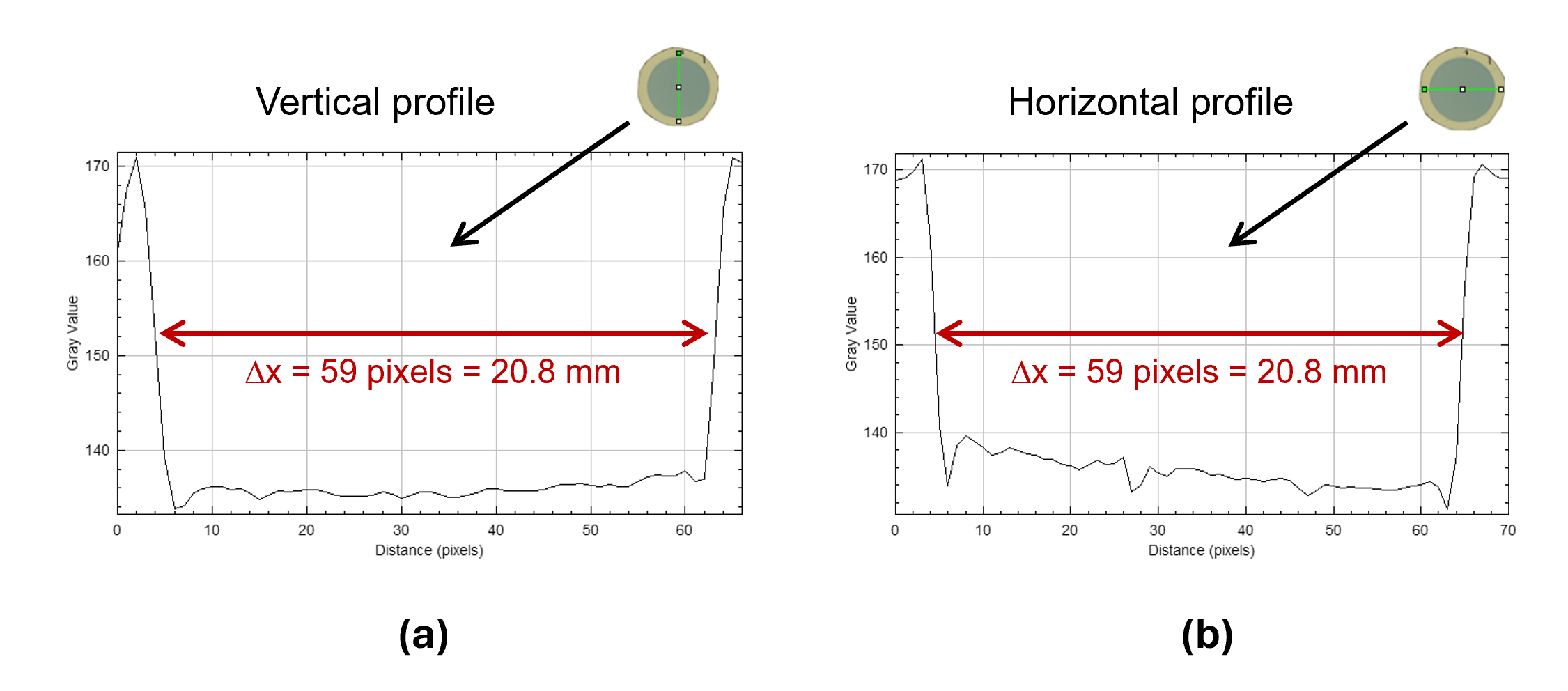


**Fig. S2.** Beam profiles (gray value as a function of distance in pixels) obtained in the Fiji software [4]. **(a)** Vertical profile and **(b)** horizontal profile with the respective lines used for quantification in the scanned images. A gap ∆x = 59 pixels was estimated for the plateau region, corresponding to a diameter of 20.8 mm in the 72 dpi setting. Mean gray values for the plateaus are 135.88 and 135.47 with maximum deviations from the mean of 2.5% and 3.9%, respectively.

## Dose rate quantification

The pulsed dose rate ($PDR)$ in our irradiation system is defined as the ratio between the film dose (D_film_) and the total pulse time (t_total_pulse_), i.e., the time sum of all individual pulses in a certain irradiation. The PDR depends on the mean dose rate (MDR), the duration of single pulse (t_pulse_) and on the frequency of the rotation disk (f) as follows

|  | $PDR (\mathrm{Gy}/{s)}=\frac{D_{film} \left( Gy \right)}{t_{total\_pulse}\left( s \right)}= \frac{MDR (\mathrm{Gy}/{min)}}{t_{\mathrm{pulse}}\left( s \right)\cdot f \left( \mathrm{rpm} \right)}=\frac{MDR (\mathrm{Gy}/{min)}}{{t_{\mathrm{pulse}}(s)}/\min}$ | **(S3)** |
| --- | --- | --- |

where the factor t_pulse_ (s)/min is the beam opening time per minute described in **Table 1**. To estimate the PDR with **Eq. S3**, intermediate steps are needed involving the determination of the total pulse time (t_total_pulse_), the mean dose rate (MDR) and the number of pulses per irradiation (N_pulse_) according to the following equations

|  | $t_{total\_pulse} \left( s \right)=N_{pulse}\cdot t_{pulse} (s)$ | **(S4)** |
| --- | --- | --- |
|  | $MDR ({Gy}/{s)}=\frac{D_{film} (Gy)}{t_{total} (s)}$ | **(S5)** |
|  | $N_{pulse}=t_{total} \left( s \right)\cdot f(s^{-1})$ | **(S6)** |

The MDR is defined as the ratio of the dose estimated with the radiochromic films (D_film_) and the corresponding total beam delivery time (t_total_), i.e., the time from beam on to beam off. Finally, the dose per pulse (DPP) is obtained by

|  | $\mathrm{DPP}\left( \mathrm{Gy} \right)=PDR \left( \mathrm{Gy}/s \right)\cdot t_{\mathrm{pulse}}(s)$ | **(S7)** |
| --- | --- | --- |

## Estimation of proton flux

In this irradiation set-up, beam current is proportional to the number of secondary electrons released from a 20 µm-thick Al foil when transversed by the proton beam. The secondary electron yield of protons incident on pure aluminum ($Y_{Al}$) is calculated with **Eq. S8**, where ${dE}/{dx}$ is the stopping power of the incident protons in aluminum in eV/cm and $E$ is the energy of the incident protons in MeV. In fact, the Al foil is composed of aluminum oxide (Al_2_O_3_) as pure aluminum oxidizes when exposed to air. The secondary electron yield of aluminum oxide ($Y_{{Al}_{2}O_{3}}$) is linearly related to the secondary electron yield of pure aluminum ($Y_{Al}$) as indicated in **Eq. S9** [5,6].

|  | $Y_{Al}=1.6\times{10}^{-9} \left( {dE}/{dx} \right)\left[ 1+\frac{1}{1+ \frac{E}{0.183}} \right]$ | **(S8)** |
| --- | --- | --- |
|  |  |  |

|  | $Y_{{Al}_{2}O_{3}}=2.25 Y_{Al}$ | **(S9)** |
| --- | --- | --- |

According to **Table S1**, the proton beam reaches the Al foil with an energy E = 15.67 MeV, whose stopping power (${dE}/{dx}$) in Al is 6.475 x 10^7^ eV/cm [1]. Thus, the secondary electron yield of pure aluminum ($Y_{Al}$) is 0.105 (according to **Eq. S8**) and, consequently, the secondary electron yield of aluminum oxide (Al_2_O_3_) is 0.236 for these conditions (according to **Eq. S9**).

In turn, the beam current ($I_{beam}$) is estimated from the secondary current read by the electrometer ($I_{sec}$) and the secondary electron yield of aluminum oxide ($Y_{{Al}_{2}O_{3}}$) (**Eq. S10**), being a key parameter to determine the number of protons per second (# protons/s) using **Eq. S11**, where *e* is the elementary charge (1.6 x 10^−19^ C). Ultimately, the proton flux ($\varphi$) is the ratio of the number of protons per unit time and the irradiated Al circular area as shown in **Eq. S12** (radius, r = 14 mm).

|  | $I_{beam}= \frac{I_{sec}}{Y_{{Al}_{2}O_{3}}}$ | **(S10)** |
| --- | --- | --- |

|  | $\# protons/s =\frac{I_{beam} \left( A \right)}{e \left( C \right)}$ | **(S11)** |
| --- | --- | --- |

|  | $\varphi({\# protons}/{\left( s\cdot{cm}^{2} \right))=\frac{{\# protons}/s}{\pi r^{2}}}$ | **(S12)** |
| --- | --- | --- |

In the end, the proton flux at the target ($\varphi_{target}$) is reduced by a factor of 5.31 x 10^-4^ due to beam scattering in the rotating disk and is related to the mean dose rate (MDR) as follows [7]:

|  | $MDR {[Gy}/{s]=1.6\times{10}^{-9}\cdot\frac{dE}{dx}\left[ \frac{keV}{\mu m} \right]\cdot\varphi_{target}\left[ \frac{1}{s\cdot{cm}^{2}} \right]\cdot\frac{1}{\rho}\left[ \frac{{cm}^{3}}{g} \right]}$ | **(S13)** |  |
| --- | --- | --- | --- |

where dE/dx is the stopping power of the proton beam at the active layer of the radiochromic film for the average energy of entrance and exit (E = 13.57 MeV, dE/dx = 4.339 keV/μm) and ρ is the active layer density (ρ = 1.2 g/cm^3^) [3]. **Table S2** presents the proton fluxes at the target ($\varphi_{target}$) and the Al foil ($\varphi_{Al})$ for MDRs of 2.0, 2.5 and 3.0 Gy/min.

**Table S2**

Proton fluxes at the target ($\varphi_{target}$) and the Al foil ($\varphi_{Al}$) for mean dose rates (MDRs) of 2, 2.5 and 3 Gy/min.

| **MDR (Gy/min)** | $\boldsymbol{\varphi}_{\boldsymbol{target}} \left( \frac{\mathbf{protons}}{\boldsymbol{s\cdot}\mathbf{cm}^{\mathbf{2}}} \right)$ | $\boldsymbol{\varphi}_{\boldsymbol{Al}} \left( \frac{\mathbf{protons}}{\boldsymbol{s\cdot}\mathbf{cm}^{\mathbf{2}}} \right)$ |
| --- | --- | --- |
| 2.0 | 5.76 x 10^6^ | 1.08 x 10^10^ |
| 2.5 | 7.20 x 10^6^ | 1.36 x 10^10^ |
| 3.0 | 8.64 x 10^6^ | 1.63 x 10^10^ |

## Clonogenic survival and the LQM model

To obtain the survival curve, the plating efficiency (PE) was calculated as the ratio of the number of colonies formed and the number of cells seeded for each dish, while the survival fraction (SF) was estimated as the ratio of the PEs of each treated condition to the controls (cells not exposed to radiation). In the end, the experimental survival curve was fitted to the linear quadratic regression model (LQM) [8] (**Eq. S14**) using the GraphPad Prism 9.0 software:

|  | $SF (D)= e^{-\alpha D-\beta D^{2}}$ | **(S14)** |
| --- | --- | --- |

In this model, the dependence of the SF on dose D is described by the parameters α and β, which characterize cellular radiosensitivity: α is the probability that a single ionizing event causes a DNA double strand break (DSB) typical of lethal damage, whereas β is the probability of several ionizing events to induce a group of DNA single strand breaks (SSB) whose overall effect results in DSBs, being associated to sublethal damage.

To further compare the biological effect of proton and photon exposure in U373 cells, Relative Biological Effectiveness (RBE) was estimated as the ratio of the photon reference dose ($r$), i.e. Co-60 γ-rays or 160 kVp X-rays, and proton dose ($p$) for the same biological endpoint (SF = 50% or SF = 10%):

|  | ${RBE}_{D_{SF}, r}=\frac{D_{SF, r}}{D_{SF, p}}$ | **(S15)** |
| --- | --- | --- |

**Table S3** presents the estimated LQM parameters for 14 MeV protons along with the previously published for photon irradiation and proton RBE values considering 160 kVp X-rays or Co-60 γ radiation as reference [9].

**Table S3**

LQM regression parameters estimated for the 14 MeV-proton experimental survival curves (Mean ± SE) and previously published experimental photon irradiation results (in gray color) adapted from [9]. Proton RBEs were calculated at D_50%_ and D_10%_ using 160 kVp X-rays or Co-60 γ-rays as reference radiation. SE is estimated by error propagation for D_50%_, D_10%_ and RBE values.

| LQM parameters | 14 MeV protons | Co-60 γ-rays [9] | 160 kVp X-rays [9] |
| --- | --- | --- | --- |
| α | 0.485 ± 0.053 | 0.181 ± 0.063 | 0.626 ± 0.059 |
| β | 0.073 ± 0.026 | 0.042 ± 0.020 | 0.020 ± 0.029 |
| α/β | 6.63 ± 3.02 | 4.28 ± 3.46 | 31.28 ± 48.77 |
| R^2^ | 0.991 | 0.940 | 0.990 |
| D_50%_ (Gy) | 1.21 ± 0.11 | 2.44 ± 0.59 | 1.07 ± 0.11 |
| D_10%_ (Gy) | 3.20 ± 0.33 | 5.54 ± 1.49 | 3.33 ± 0.50 |
| RBE_50%, Co-60 γ-rays_ | 2.02 ± 0.52 | -------------- | -------------- |
| RBE_50%,160 kVp X-rays_ | 0.89 ± 0.12 | -------------- | -------------- |
| RBE_10%, Co-60 γ-rays_ | 1.73 ± 0.50 | -------------- | -------------- |
| RBE_10%,160 kVp X-rays_ | 1.04 ± 0.19 | -------------- | -------------- |

## DNA damage quantification

At 1 h and 24 h after radiation exposure, U87 cells were rinsed with PBS and fixed with absolute ethanol for 20 min at room temperature. After a second wash with PBS, cell permeabilization was performed for 15 min with Triton X-100 (0.2% in PBS, Sigma-Aldrich). Subsequently, the cells were rinsed with PBS and blocked with BSA (3% in PBS, Sigma-Aldrich) for 1 h, being then incubated with the monoclonal primary antibody γH2AX (rabbit, 1:250, cat.no. ab81299, Abcam) overnight at 4 ºC in a humidified chamber. On the next day, the samples were rinsed 3 times with PBS and incubated with anti-rabbit Alexa Fluor 647 (1:200, cat.no. A21245, Invitrogen) stained for nuclei assessment with 2 mg/ml Hoechst 33,342 (1:1000, Sigma-Aldrich) for 1 h protected from light. Both antibody solutions included 5% Triton-X and 1% BSA as well as PBS until desired volume. After that, 15 mm-glass inserts were added to each well along with Dako Fluorescence Mounting Medium (Agilent Technologies) and left to dry. Fluorescent images were acquired in an Axio Observer Z1 inverted microscope (Carl Zeiss, 20x magnification) and quantification was performed in the Cell Profiler software [10], where total γH2AX fluorescence intensity of segmented nuclei was assessed. For each condition, 3 image fields with at least 100 cells were quantified in a total of 3 independent experiments performed (N=3). Statistical analysis was performed in GraphPad Prism 9 and included a two-way ANOVA followed by Tukey’s multiple comparisons test to identify statistically significant differences considering a 95% confidence interval (threshold p-value = 0.05).

## Cell culture details

The human glioblastoma cell line U87 was obtained from the American Type Culture Collection (ATCC), while U373 Uppsala cells were purchased from the European Collection of Authenticated Cell Cultures (ECACC). Both cell lines were cultured at 37 ºC in a 5% CO_2_ atmosphere using Minimum Essential Medium (MEM) Eagle with Glutamax supplemented with 10% Fetal Bovine Serum (FBS) and 1% penicillin/streptomycin. U373 cells required extra supplementation with 1% MEM non-essential amino acids and 1% sodium pyruvate. Both cell lines were authenticated by Eurofins and tested with the LookOut® mycoplasma Polymerase Chain Reaction (PCR) Detection kit (Sigma-Aldrich), which confirmed they were free of mycoplasma contamination.

# References

[1] Ziegler J. SRIM - The Stopping and Range of Ions in Matter 2013. http://www.srim.org/ (accessed December 9, 2025).

[2] HPM – Hamilton Precision Metals. Technical data sheet – Havar n.d. https://www.hpmetals.com/-/media/ametekhpmetals/files/technical-data/ni-base-corrosion-resistant/havar.pdf (accessed February 26, 2026).

[3] Palmer AL, Dimitriadis A, Nisbet A, Clark CH. Evaluation of Gafchromic EBT-XD film, with comparison to EBT3 film, and application in high dose radiotherapy verification. Phys Med Biol 2015;60:8741. https://doi.org/10.1088/0031-9155/60/22/8741.

[4] Schindelin J, Arganda-Carreras I, Frise E, Kaynig V, Longair M, Pietzsch T, et al. Fiji: an open-source platform for biological-image analysis. Nature Methods 2012 9:7 2012;9:676–82. https://doi.org/10.1038/nmeth.2019.

[5] Borovsky JE, McComas DJ, Barraclough BL. The secondary-electron yield measured for 5–24 MeV protons on aluminum-oxide and gold targets. Nucl Instrum Methods Phys Res B 1988;30:191–5. https://doi.org/10.1016/0168-583X(88)90116-4.

[6] Castaneda CM, McGarry L, Cahill C, Essert T. Secondary electron yields from the bombardment of Al2O3 by protons, deuterons, alpha-particles and positively charged hydrogen molecules at energies in the range of 10 to 80 MeV. Nucl Instrum Methods Phys Res B 1997;129:199–202. https://doi.org/10.1016/S0168-583X(97)00282-6.

[7] Ghithan S, Crespo P, Carmo SJC do, Marques RF, Fraga FAF, Simões H, et al. Development of a PET cyclotron based irradiation setup for proton radiobiology. Journal of Instrumentation 2015;10:P02010–P02010. https://doi.org/10.1088/1748-0221/10/02/P02010.

[8] McMahon SJ. The linear quadratic model: Usage, interpretation and challenges. Phys Med Biol 2019;64. https://doi.org/10.1088/1361-6560/AAF26A.

[9] Teixeira ARC, Antunes J, Pinto CIG, Campello MPC, Santos P, Gomes CM, et al. GRPR-targeted gold nanoparticles as selective radiotherapy enhancers in glioblastoma. Phys Med Biol 2025;70:125018. https://doi.org/10.1088/1361-6560/ADE222.

[10] Stirling DR, Swain-Bowden MJ, Lucas AM, Carpenter AE, Cimini BA, Goodman A. CellProfiler 4: improvements in speed, utility and usability. BMC Bioinformatics 2021;22:1–11. https://doi.org/10.1186/S12859-021-04344-9/FIGURES/6.
